# GOssTo: a stand-alone application and a web tool for calculating semantic similarities on the Gene Ontology

**DOI:** 10.1093/bioinformatics/btu144

**Published:** 2014-03-22

**Authors:** Horacio Caniza, Alfonso E. Romero, Samuel Heron, Haixuan Yang, Alessandra Devoto, Marco Frasca, Marco Mesiti, Giorgio Valentini, Alberto Paccanaro

**Affiliations:** ^1^Department of Computer Science, Royal Holloway, University of London, Egham, TW200EX, UK, ^2^School of Mathematics, Statistics & Applied Mathematics, National University of Ireland, Galway, ^3^School of Biological Sciences, Royal Holloway, University of London, Egham, TW200EX, UK and ^4^Dipartimento di Informatica, Università degli Studi di Milano, via Comelico 39/41, 20135 Milano, Italy

## Abstract

**Summary:** We present GOssTo, the Gene Ontology semantic similarity Tool, a user-friendly software system for calculating semantic similarities between gene products according to the Gene Ontology. GOssTo is bundled with six semantic similarity measures, including both term- and graph-based measures, and has extension capabilities to allow the user to add new similarities. Importantly, for any measure, GOssTo can also calculate the Random Walk Contribution that has been shown to greatly improve the accuracy of similarity measures. GOssTo is very fast, easy to use, and it allows the calculation of similarities on a genomic scale in a few minutes on a regular desktop machine.

**Contact**: alberto@cs.rhul.ac.uk

**Availability:** GOssTo is available both as a stand-alone application running on GNU/Linux, Windows and MacOS from www.paccanarolab.org/gossto and as a web application from www.paccanarolab.org/gosstoweb. The stand-alone application features a simple and concise command line interface for easy integration into high-throughput data processing pipelines.

## 1 INTRODUCTION

Semantic similarity measures have become important in bioinformatics as they provide a way of quantifying the functional relatedness between genes that is complementary to both experimental information and sequence-based approaches. This is done by annotating genes to the terms of a chosen ontology and then quantifying the similarity between these terms. Among the ontologies, the Gene Ontology (GO) (Ashburner *et al.*, 2000) has become a standard and is the focus of this work.

Several semantic similarity measures have been proposed. For example, those by Resnik (1999), Jiang and Conrath (1997) and Lin (1998) are based on the information content of the lowest common ancestor of pairs of terms, and are often referred to as ‘term-based’; simUI and simGIC (Pesquita *et al.,* 2008) and GraSM (Couto and Silva, 2005) compare sets of terms rather than single terms using graph comparison approaches and are often referred to as ‘graph-based’.

An important recent development has been the introduction of the Random Walk Contribution which greatly improves semantic similarity measures (Yang *et al.*, 2012). In their paper, the authors argued that existing measures have two important deficiencies: first, they do not take into account the descendants of the terms; second, they do not model the inherent uncertainty in the current annotations and ontology structure. The Random Walk Contribution is a kind of ‘add on’ for existing similarity measures that enhances them to correct these two issues.

Few software tools have been proposed for calculating semantic similarities, including ProteinOn (Faria *et al.,* 2007) IT-GOM (Mazandu and Mulder, 2013) and G-SESAME (Du *et al.,* 2007). However, none of them can combine the Random Walk Contribution proposed by Yang *et al.* (2012). Moreover, tools provided as stand-alone applications are not readily extendable with new semantic similarity measures, or are available only as packages running within environments such as R or MATLAB. Other tools are exclusively available online and their use is impractical for high-throughput analysis on large bodies of data. Most tools do not allow for a straightforward calculation of semantic similarities for a whole genome, or an easy updating of the GO annotations.

In this article, we present GOssTo (Gene Ontology semantic similarity Tool), a new tool for calculating semantic similarities that overcomes all of the above limitations. GOssTo includes the Random Walk Contribution by Yang *et al.* (2012) and it supports both term- and graph-based similarity measures.

GOssTo is available in downloadable binary form, with the entire source code released under GPLv3. GOssTo is easy to use and very fast—Table 1 shows the time required for calculating the Resnik semantic similarity including the Random Walk Contribution for a few model organisms. GOssTo features a simple and concise command line interface and an application programming interface (API) for easy integration into high-throughput data-processing pipelines. GOssTo’s design allows for user provided similarity measures to be independently developed, compiled and linked at runtime. These features make GOssTo a practical environment for both the development of novel semantic similarity measures as well as for the calculation of semantic similarities on a genomic scale.

**Table 1.** Time, in minutes, required for calculating semantic similarities for a few model organisms

Organism	Number of GO terms	Number of annotated genes	Time term-wise	Time gene-wise
Arabidopsis	6610	9703	3 m 48 s	43 m 35 s
Rat	9422	5270	58 m 19 s	29 m 54 s
Mouse	12961	15020	24 m 35 s	689 m 26 s
Fly	7304	8235	4 m 56 s	47 m 46 s
Yeast	7077	4898	4 m 0 s	23 m 55 s
Worm	4467	4370	1 m 29 s	5 m 1 s

*Note*: For each organism: number of unique GO terms appearing in the GO annotation; number of annotated genes; time (in minutes and seconds) required for calculating the Resnik semantic similarity including the Random Walk Contribution term- and gene-wise. Calculations used GO experimental evidence codes (EXP, IDA, IPI, IMP, IGI, IEP, TAS) and *is_a* and *part_of* GO relationships. Data downloaded in February 2014. Experiments run on AMD Opteron 6128 HE.

GOssTo is also available online, through a clean web interface from our server at www.paccanarolab.org/gosstoweb. GOssToWeb provides access to the same functionalities of the stand-alone application, allowing extensive configuration of the experiments through a user-friendly web form. The user can select GO evidence code, GO relationships and a genome from the list of organisms available in UniProt-GOA. GOssToWeb automatically fetches the most recent version of the functional annotation from UniProt-GOA and of the GO from its official repository, thus ensuring that the most up-to-date data are used. Results are provided by redirecting the user to a page from which they can be downloaded. The system can notify the user with an email containing a link to the result download page.

## 2 METHODS

The downloadable version of GOssTo is bundled with six commonly used semantic similarity measures: the term-based measures by Resnik (1999), Lin (1998), Jiang and Conrath (1997) and GraSM (Couto and Silva, 2005); the graph-based measures simUI and simGIC (Pesquita *et al.,* 2008). All these measures are extended with Yang *et al.* (2012) Random Walk based procedure.

The guiding principles for GOssTo’s design aimed at producing a fast and flexible software package. This resulted in a highly modularized architecture with very low coupling between individual modules. These modules can be readily removed or replaced without affecting the overall behaviour of the system.

The user can interact with GOssTo either through a command-line interface or an API. The command-line interface provides UNIX-like console parameter options as well as an interactive menu; the API offers access to all the functionalities in the different modules through a set of well-defined functions. Thus, GOssTo can be used in three different ways: as a part of a larger data-processing pipeline; as a stand-alone application; as a static library for existing software. For easy processing of the results, all output is presented in structured plain text files.

GOssTo includes a powerful extension mechanism to add new semantic similarity measures. A well-defined interface grants the user access to the data structures upon which new measures can be developed. After a new measure is independently compiled, it can be dynamically linked to GOssTo’s application core. The new measure can then be used in the same way as the ones bundled with GOssTo. The current version of GOssTo focuses on traditional semantic similarity measures which rely mostly on the GO structure. Future versions will include the possibility of handling Description Logic axioms which are being added to existing ontologies (Ferreira *et al.*, 2013).

GOssTo was developed using the Java programming language. The JAMA package provides the internal data types and the required mathematical routines. GOssTo’s source code is freely available from GitHub at https://github.com/pwac092/gossto and is released under the GPLv3 license. GOssTo runs on multiple platforms, and we have extensively tested in on both GNU/Linux and Windows. More information about GOssTo including a comprehensive manual is available from www.paccanarolab.org/gossto

*Funding*: Biotechnology and Biological Sciences Research Council (BBSRC) (grant number BB/K004131/1 to A.P.); PASCAL2 Network of Excellence (EC grant number 216886 to G.V.).

*Conflicts of Interest*: none declared.
